# Caesarean section in women following an abdominal myomectomy: a choice or a need?

**Published:** 2020-05-07

**Authors:** F Odejinmi, S Strong, M Sideris, R Mallick

**Affiliations:** Whipps Cross Hospital, Barts Health NHS Trust, Whipps Cross Road, Leytonstone, London, E11 1NR;; Princess Royal Hospital, Brighton and Sussex University Hospitals NHS Trust, Lewes Road, Haywards Heath, RH16 4EX.

**Keywords:** Vaginal delivery, caesarean section, fibroids, myomectomy, uterine rupture

## Abstract

Delivery options following both open and laparoscopic myomectomy remains a controversial topic and opinions vary between obstetricians and gynaecologists. The historical advice of planned caesarean section before 39-weeks persists despite the movement towards the minimal access approach for myomectomy. The main concern remains the small, but potentially catastrophic risk of uterine rupture. Unfortunately, there remains a paucity of data assessing factors that can affect the uterine integrity following laparoscopic myomectomy, such as number, size and type of fibroids, uterine cavity breach and electro-cautery usage. Despite this, the cited 1% overall risk of rupture following myomectomy is similar to the quoted risk following trial of labour after caesarean section, and a successful and safe vaginal delivery can be achieved in as high as 90%. Patient choice and informed consent are essential in the holistic approach to managing these women and safely supporting their delivery choices.

## Introduction

Fibroids are the commonest benign tumour of the female genital tract, affecting up to 80% of women (Baird et al., 2003). They are symptomatic in 25- 30% of women and most symptomatic women seek treatment prior to pregnancy (Brito et al., 2014). Despite advances in medical management, myomectomy remains the commonest surgical procedure for women who opt to have their fibroids removed, but want to preserve their uterus and subsequent fertility. Though myomectomy can improve symptoms related to fibroids, the scars left on the uterus can affect quality of life and have consequences for both subsequent conception and delivery. Uterine rupture is a potentially catastrophic obstetric emergency for both mother and baby that can result from a scarred uterus secondary to a previous myomectomy.

In view of this, the American College of Obstetrics and Gynaecology (ACOG) currently recommends an elective caesarean section for women who have had a previous myomectomy where the endometrial cavity is entered (Goetzl, 2002). Although this is understandably precautionary, is there sufficient evidence to completely support this view? The aim of this article is to review the current evidence, quantify the risks of a previous uterine scar after open and laparoscopic myomectomy and assess factors which may guide the safest mode of subsequent delivery.

## Background

A previous caesarean section is probably a more common reason for uterine rupture, however when conducted diligently the risk of uterine rupture is relatively rare. Over 100 years ago, women who had a previous caesarean section were advised to have elective caesareans in all subsequent pregnancies, based on the view of that time, highlighted by the statement “once a caesarean always a caesarean” coined by Edward Cragin (Foster, 2017). This view has subsequently changed over time, tempered by emerging safety evidence balancing the risk of uterine rupture against the risks of multiple caesarean sections (Friedman, 2019) and taking women’s choice into consideration. Furthermore this work is still ongoing and constantly evolving with the development of tools to that help predict the risk of uterine rupture (Smith et al., 2005). Unfortunately, to date, no such “tools” exist for women who have had a previous myomectomy and advice is often based on historical data.

## The risk of uterine rupture following open and laparoscopic myomectomy

Before the widespread use of minimal access surgery for the surgical management of uterine fibroids, the risk of uterine rupture after myomectomy was historically estimated at 2.5% (Georgakopoulos and Bersis, 1981). This was probably much of an overestimate and obstetricians used clinical judgement, often regarding a breach of the uterine cavity as the greatest risk factor for subsequent rupture, always advising early caesarean sections in such cases (Landon and Lynch, 2011). In order to mitigate against the possible risk of rupture, delivery by caesarean was often deemed best undertaken before 39 weeks gestation. The timing is likely an extrapolation of the findings of James Garnet (1964), who observed that uterine ruptures occurred 1 to 2 weeks before term in twothirds of women with a previously scarred uterus. Others postulated that all women with previous myomectomies should be treated as if they had undergone a classical caesarean section (Al Qahtani, 2013) and delivery options guided accordingly.

Since the early 1980s, when the first laparoscopic myomectomy was performed, and with the advancement of surgical skill and technology, the gold standard for most women undergoing a myomectomy has been the laparoscopic approach (Mallick and Odejinmi, 2017). Furthermore, the benefits of using the laparoscopic approach to tackle large submucous fibroids are becoming clearer (Oxley et al., 2019). However, this results in a planned breach of the uterine cavity, which historically would have been a complete contra- indication to vaginal delivery.

The movement towards the laparoscopic approach has increased the spotlight on the issue of uterine rupture after previous myomectomy. Parker and colleagues reviewed 19 cases of uterine rupture occurring after previous laparoscopic myomectomy, reported in literature over an 18 year period and found no single risk factor that contributed to uterine rupture (Parker et al., 2010). They concluded that if the technique used for open myomectomy is the same for laparoscopic surgery, previous laparoscopic myomectomy per se should not increase the risk of uterine rupture over laparotomy. This view can be further supported by studies looking at uterine scar integrity after laparoscopic compared to open myomectomy where no difference in ultrasonic integrity of uterine scars following either procedure was found (Asgari et al., 2019).

In a systematic review of the incidence of uterine rupture following myomectomy, Claeys et al. (2014) estimate the risk at 0.79% during labour (1.2% following laparoscopic myomectomy and 0.4% following open surgery), whilst Nahum and Pham (2012) put the risk at 0.7% (1.7% after abdominal myomectomy and 0.49% after laparoscopic myomectomy). This is comparable to a 1% rupture rate following previous caesarean section and concludes that women should not be discouraged from attempting vaginal delivery after myomectomy whether laparoscopic or open.

Unfortunately for those women who suffer uterine rupture, the majority do so before the onset of labour. Taking this into consideration, for women who go into labour and have a well conducted trial of labour, the risk of rupture is 0.47% (Gambacorti- Passerini et al., 2016).

Thus the overall rupture risk of approximately 1% is similar to the quoted rupture risk following trial of labour after caesarean section, and is further supported by the success of vaginal delivery after myomectomy, which can be as high as 90% without uterine rupture or severe maternal morbidity (Gambacorti-Passerini et al., 2016). This rupture risk of less than 1% in labour should be weighed against the potential morbidity of caesarean delivery after myomectomy. Gimovski et al. (2018) compared 33,365 control patients with 367 women who had previously undergone a myomectomy and required a caesarean section. They reported a significantly higher risk of complications at the time of caesarean section, such as higher rates of intraoperative and postoperative transfusions, bowel injury, caesarean hysterectomy and the need for a classical uterine incision.

## Factors that could affect the risk of rupture after a myomectomy

The above demonstrates that there is conflicting evidence on how women who have had myomectomies should be managed in labour. If evidence is to be gathered, it needs to be around parameters that could affect the integrity of the uterus post myomectomy:

1. Number of fibroids removed
2. The number of uterine incisions
3. The size of the fibroids removed
4. The location of the fibroids removed
5. Technology used for haemostasis (either electrocautery or modern ultrasonic devices).
6. Type of suture used and number of layers of closure
7. Haematoma development after myomectomy

Other postulations affecting the risk of rupture include individual patient characteristics and the use of pneumoperitoneum during laparoscopic procedures (Mynbaev et al., 2016).

To date, however, there are no studies in the wider literature that have addressed these issues individually. On the issue of size of fibroids and scar integrity, Seinera et al. (1999) performed uterine artery dopplers and ultrasound follow up, and found no difference in scar integrity irrespective of the size of myoma removed by day 30 post- operatively. Extrapolation would thus infer that the size of fibroids removed should not affect the integrity of the scar and therefore should not affect the risk of rupture. Although Parker et al. (2010) found no single risk factor that contributed to uterine rupture, they postulated that haematoma formation may have a detrimental effect on wound healing and uterine integrity. Controversy exists around single and multi-layer uterine closure, both in the context of myomectomy and caesarean section. However, extrapolation from caesarean section data would suggest a single layer closure may increase the rate of uterine rupture (Gyamfi et al.,2006). National guidance also advises a two layer closure (NICE, 2011).

There remains a paucity of data regarding other factors that may affect uterine integrity, and hence the risk of uterine rupture, such as the number of fibroids removed, the number of incisions used when removing fibroids, the energy devices used and suturing technique/material used. Further independent studies are required to assess such factors to guide practice, aid informed patient consent and facilitate shared decision making.

In order to be able to quantify the risk fully, uterine rupture after myomectomy should be a reportable incident, with central databases kept. For further insight, standardised operation proformas for myomectomy should also be developed, as this will allow for comparison of complexity of the surgery, and may also point to risk and help aid patient consent.

## How clinicians currently make decisions

A recent Canadian study on the perspectives of 49 Canadian obstetricians suggests that women who have had a myomectomy with a breach of the uterine cavity should be prevented from having a vaginal delivery (Weibel et al., 2014). The issue of entry into the uterine cavity was also observed in a retrospective review, where there was a significant difference in the number of women undergoing elective caesarean sections compared to planned vaginal delivery (Gambacorti-Passerini et al., 2018). The above review however does not support breach of the uterine cavity as the only reason for potential rupture.

## What do women want?

There have been previous studies in the area of caesarean section and preferred methods of delivery (McGurgan et al., 2001), and also studies that suggest that women should be counselled individually on the risks and benefits of caesarean sections in line with NICE guidelines (Aref-Adib et al., 2018). To date, however, there are no such studies on preferences of women who have had a myomectomy.

## Medicolegal considerations

To date there is no data looking at the medicolegal issues surrounding uterine rupture following myomectomy or rupture during attempted vaginal delivery. If extrapolations are made from the trial of labour after caesarean section, malpractice liability needs to be considered (Friedman, 2019) taking the previously stated evidence of success of vaginal delivery after myomectomy, (Gambacorti-Passerini et al., 2018) coupled with women’s choice.

Careful selection of patients who want to attempt a vaginal delivery who have a clear understanding of the risk factors will help improve patient safety (Vandenberghe et al., 2019), and should obviate litigation risk as with previous caesarean sections.

## Conclusion

Unfortunately, the true risk of uterine rupture after myomectomy remains unknown, as most systematic reviews report only on the small case series available in the wider literature. Most of these case series do not report on all women who achieve pregnancy, and uterine rupture may go unreported as many women may present to hospitals other than those where the original myomectomy was performed.

On the basis of this available evidence, there is very little to guide what increases the risk of uterine rupture such as whether the uterine cavity is entered or not, and on balance the risks of a vaginal delivery may outweigh the risks of a caesarean section in women who have had a previous myomectomy. However, for many women, vaginal delivery is a safe and feasible option. Patient choice and informed consent is thus key. Women should be offered a choice, counselled appropriately using all available evidence, and managed in units that support their choice.

Ongoing national audit reporting delivery outcomes following myomectomy are crucial to ensuring that women can be supported to make informed, safe choices. One further step towards gathering data would be standardised surgical proformas for uterine fibroids, detailing key operative details and patient characteristics, and the development of risk assessment tools. Uterine rupture of any sort should be a reportable pregnancy outcome, thus allowing clinicians and women to further understand factors associated with the risk of rupture, and again support informed patient consent.
